# Accidental Chlorine Gas Intoxication: Evaluation of 39 Patients

**DOI:** 10.4021/jocmr2009.12.1283

**Published:** 2009-12-28

**Authors:** Mustafa Sever, Cengiz Mordeniz, Fidan Sever, Mehmet Dokur

**Affiliations:** a Department of Emergency Medicine, Harran University School of Medicine, Sanliurfa, Turkey; b Department of Anesthesiology and Reanimation, Harran University School of Medicine, Sanliurfa, Turkey; c Pulmonary Medicine Ward, Sifa Bornova Medical Center, Izmir, Turkey; d Emergency Service, Kilis State Hospital, Kilis, Turkey

## Abstract

**Background:**

Chlorine is a known pulmonary irritant gas that may cause acute damage in the respiratory system. In this paper, the socio-demographic and clinical characteristics of 39 accidentally exposed patients to chlorine gas are reported and different emergency treatment modalities are also discussed.

**Methods:**

Two emergency departments applications were retrospectively analyzed for evaluation of accidental chlorine gas exposure for year 2007. Patients were classified into 3 groups according to severity of clinical and laboratory findings based on the literature and duration of land of stay in the emergency department. The first group was slightly exposed (discharged within 6 hours), second group moderately exposed (treated and observed for 24 hours), and third group was severely exposed (hospitalized). Most of the patients were initially treated with a combination of humidified oxygen, corticosteroids, and bronchodilators.

**Results:**

The average age was 17.03 ± 16.01 years (95% CI). Seven (17.9%) of them were female and 29 (74.4%) were children. Twenty-four patients (61.5%) were included in the first, nine (23.1%) were in second and six (15.4%) were in the third group. The presenting symptoms were cough, nausea, and vomiting and conjunctiva hyperemia for the first group, first groups symptoms plus dyspnea for the second group. Second groups symptoms plus palpitation, weakness and chest tightness were for the third group. Cough and dyspnea were seen in 64.1% and 30.8% of the patients respectively. No patients died.

**Conclusions:**

The authors recommend that non symptomatic or slightly exposed patients do not need any specific treatment or symptomatic treatment is sufficient.

**Keywords:**

Accidental; Chlorine exposure; Chlorine gas; Chlorine intoxication; Emergency department

## Introduction

Chlorine (Cl_2_) is a yellow-green gas or boiling yellow liquid, slightly water soluble, and about two times heavier then air [1,2]. It has a pungent, irritating odor and is a strong oxidizing agent [1,2]. Chlorine releasing agents are frequently used for industrial, household chemicals and water purification in swimming pools or city water sources [2-4].

The risk of exposure to chlorine gas with large number of casualties is widespread, since chlorine is carried through densely populated areas in large quantities [3]. Inhalation exposure to chlorine can occur from inhalation of elemental Cl_2_ or inspiration of vapors that contain chlorine releasing chemicals [4-7] due to chemical transportation accidents [8,9], accidental explosions, leaks, or malfunction of chlorine-disinfection systems [7], improper mixing of ammonia and hypochlorite bleach (forming chloramines gas) and school experiments [1,8,10].

The respiratory system is the most adversely affected of all organ systems by chlorine gas exposure [11] and complications are generally immediate with severe exposure, including acute respiratory distress syndrome (ARDS), respiratory failure, pneumomediastinum and death [1,8,12-16].

In this paper, the social and demographic characteristics, clinical and laboratory findings of patients which have been accidentally exposed to chlorine gas due to chlorine tank explosion in Southeast of Turkey are reported. Different treatment modalities in chlorine intoxication are also discussed.

## Patients and Methods

There are four hospitals in the city center which the study is performed. Three of them are Ministry of Health Government Hospitals (one is Children’s Hospital) and other is University Hospital. All hospitals’ emergency departments (ED) medical records which explain above were retrospectively analyzed for evaluation of accidental chlorine exposure patients for year 2007.

The authors determined totally 168 people who had been affected acutely by chlorine gas as a result of chlorine tank explosion that was used for city water source purification, and transferred to the hospitals in the city center. Among these, authors could reach only 39 patients’ medical records (Twenty three patients transferred to Ministry of Health Children’s, 16 to University Hospital EDs). The rest of 129 patients medical records which transferred to other two hospitals EDs were not able to reach because the reasons of cant get permission and file record insufficiency.

The authors determined 39 patients’ routine physical examinations were done at admission. Depending on the patients clinical picture by the discretion of the physician, the necessary diagnostic tests (e.g. chest X-Ray (CXR)), complete blood counts, electrocardiograms (ECG), arterial blood gases (ABG), and consultations were ordered.

The authors classified the patients into 3 groups according to clinical and laboratory findings based on the literature [1, 8, 12-14, 16] and duration of land of stay in the ED. First group was not affected at all or slightly exposed. These patients were principally mobile, they showed no vital or laboratory abnormalities. The main symptoms were mainly mild cough, nausea, weakness and burning in the eyes, and throat. They were completely treated and discharged from ED in less than 6 hours. The second group was moderately exposed. The main predictors of this group were mild pulmonary symptoms (as mild dyspnea and/or mild to moderate cough, and palpitation) and/or tachycardia and/or tachypnea (> 18 to ≤ 25 breath/per minute). But they had no pathologic pulmonary examination findings. This group of patients were treated and observed in ED for 6 to 24 hours. The third group was severely exposed. They had severe dyspnea, chest tightness or pain, wheezing, tachypnea (≥ 26 breath/minute), tachycardia and pathologic pulmonary physical examination findings such as crackles and/or ronki. All patients in the third group were hospitalized. Some of the patients in the second and third groups showed abnormal laboratory results.

Most of the patients were initially treated with supplemental humidified oxygen (via nasal canulla or mask). Various combinations of parenteral fluids, metyl-prednisolone, antiemetics and/or H_2_ blockers, antibiotics and inhalational bronchodilators were used appropriately. None of the patients received nebulized sodium bicarbonate therapy.

All the data that we have presented are mean ± SD and in percentages.

## Results

Seven (17.9%) patients were females and 32 (82.1%) were males. The ages of the patients ranged between 2 and 66 years and the average age was 17.03 ± 16.01 (CI 95%). Twenty nine (74.4%) were children (ranging between 2 - 16 years of age). Thirty two patients (82.1%) had no history of medical disease or smoking habit. However, 6 patients (12.8%) were smokers, one had history of hypertension, and one child (2 years old) had asthma history. Patient characteristics according to age groups are shown in Table 1.

**Table 1:** Patient Characteristics According to Age Groups

	Children 29 (74.4)			Adults 10 (25.6)				
Age Groups	1 - 6	7 - 18		19 - 29	30 - 39	40 - 49	≥ 50	Total
(years)								
Male	9 (23.1)	14 (35.9)		1 (2.6)	3 (7.7)	3 (7.7)	2 (5.1)	32 (82.1)
Female	2 (5.1)	4 (10.3)		0	1 (2.6)	0	0	7 (17.9)
Total	11 (28.2)	18 (46.2)		1 (2.6)	4 (10.3)	3 (7.7)	2 (5.1)	39 (100)

Data are presented as “n (%)”

The presenting symptoms were mostly associated with one another, including; cough (alone) (n = 7, 17.9%), cough and nausea (n = 5, 12.8%), cough and dyspnea (n = 5, 12.8%), and different associations of these with chest tightness, burning in eyes and in throat, conjunctival hyperemia, weakness and vomiting symptoms were found in 27 (69.2%) patients respectively. Cough and dyspnea were the most prevalent complaints among the patients and were seen in 64.1% and 30.8% of the patients respectively, in total. Twelve patients (30.8%) had no evident symptoms. In physical examination 25 patients (64.1%) were normal. Tachypnea was the dominant sign with 35.9% among the patients.

Regarding the laboratory findings, 11 (28.2%) patients were assessed by CXR, 10 (25.6%) were assessed by ECG and 17 (43.6%) were assessed by ABG analyses. Most patients did not have an ECG, ABG or CXR performed. These tests were considered unnecessary for the patients who appeared well. Only one patient (2.6%) who used to be a smoker, showed pathologic findings in his chest radiograph (hyper-aeration, decrease in cardio-thoracic ratio, elevated diffuse reticular density). No ECG pathology was detected and 11 (28.2%) of ABG analyzes were abnormal. Eight (20.5%) patients, whose ABG analyzes were assessed as abnormal, had hypoxia. Four of these 8 patients were in the third group. Of these patients, one (2.6%) had respiratory alkalosis, while the rest three (7.7%) had hypercarbia and respiratory acidosis.

The authors did not need to perform decontamination to any patient because chlorine is a volatile gas and there were no severe eye or skin contaminations in our series. No patients received nebulized sodium bicarbonate therapy.

Among the patients who were evaluated in EDs, 24 (61.5%) patients were in the first group and they were discharged after initial examination and symptomatic treatment except 3 patients who refused any treatment except normal saline. Seven (17.8%) of the rest 21 patients in the first group received only humidified oxygen and metyl-prednisolone.

Other 14 (36%) patients in the first group who accepted treatment were initially treated with supplemental humidified oxygen (via nasal canulla or mask), various combinations and doses of parenteral fluids (e.g. normal saline, 0.45 % saline + 5 % dextrose, and Isolyte P^®^), and metyl-prednisolone (1mg/kg, intravenously). Of these 14 patients, 2 received metoclopramide (alone, intravenously), 3 received ranitidine (alone, intravenously) another 3 received ranitidine and metoclopramide combination (intravenously), appropriately, in addition to the initial treatment.

Nine (23.1%) patients were in the second group. In this group one patient received only humidified oxygen and metyl-prednisolone. Three patients were initially treated in the same way with the first group. In this group, in addition to initial treatment, one patient received inhaler salbutamol, 2 patients received ranitidine, and one patient received metoclopramide plus ranitidine and inhaler salbutamol combination. Another patient received initial treatment plus metoclopramide plus ranitidine and inhaler salbutamol combination and wide spectrum antibiotic (intravenously). They were all observed and discharged in 24 hours after application to ED.

There were 6 (15.4%) patients in the third group who were severely exposed were hospitalized; 2 patients were admitted to the adult intensive care unit, 3 patients to the pediatric intensive care unite and 1 patient to the pulmonary medicine department. No patient was diagnosed as bronchitis, ARDS and/or acute lung edema. Invasive or non invasive airway support (as intubations, CPAP or BIPAP) was not necessary. All the patients in this group were initially treated as in the first group. Metoclopramide, ranitidine, inhaler salbutamol and antibiotic measure combination were also used appropriately. Only one patient received teophyline measure in addition to this combination. No patients died.

## Discussion

Chlorine is a highly toxic gas well known for mortality and morbidity since its discovery in 1772 [16]. The basic mechanism of toxicity is related to the solubility of chlorine in the water, because elemental Cl_2_ maintains equilibrium with hydrochloric acid (HCl) and hypochlorous acid (HOCl) in aqueous solution (Cl_2_ + H_2_O↔HCl + HOCl) or (Cl_2_ + H_2_O↔2 HCl + [O^-^] ) [4,5]. These chemical end-products cause damage to cellular proteins, resulting in direct tissue injury [17].

The medical literature is replete, from household or swimming pool exposures to industrial or chemical transportation accident reports [2,4, 6, 9, 12, 13-16]. Most unintentional exposures occur in the industrial field [5,9,16]. Transportation mishaps can release large quantities of Cl_2_ gas into the air and trigger mass disaster incidents that can affect large number of adults and children together [9,18]. The wide availability of chlorinated compounds as household disinfectants and swimming pool chlorinators makes these agents potentially hazardous to a larger segment of the population, including children [4, 6-9].

In most of the reports, patients’ ages ranged similarly but pediatric population was not as crowded as in our series [9,15,19]. In a review of 216 patients who were exposed to chlorine gas, Mrvos et al [19] reported that they were between 12 and 81 years old. Also Guloglu et al [9] reported that population age range in their study was between 3 months and 75 years of age and 50.9% of the cases were children and adolescents. Agabiti et al [15] report was similar with Mrvos et al and Guloglu et al. They reported 282 patients and 134 (47.5%) were children.

In the present study, children’s population ratio is high because our data include the children who were transferred to Ministry of Health Children’s Hospital. The main accident involved 129 more patients, mostly adults, transferred to other hospitals in the same city. Children/adult ratio would be different if all the victims were included in this study.

Asthma, smoking habit, atopic persons and chronic exposure to chlorine gas were reported to be predisposing factors or conditions worsening the scene in many reports [8,9,11,16,18,20]. In the presented series, there were only six smokers and one asthmatic child. Three of the smokers and the asthmatic child were admitted to intensive care unit but these patient numbers were not enough for evaluating these factors.

The authors do not know how much chlorine gas was released into the atmosphere when the chlorine tank exploded. With considerable consistency around the world, chlorine has a time-weighted average exposure standard of 0.5-1 parts per million (ppm) [11], but Evans [16] reported that doses below the 0.5 ppm can cause tickling of the nose and throat, itching of the nose and cough, burning of the eyes, and dryness of the throat. The fatal dose ranges from 50 to 2,000 ppm. Although, different chlorine gas exposure ratios can cause different complaints [16].

Evans [16] also reported that even upper airway exposure from chlorine inhalation was predominant. However, exposures to concentration greater than 15 ppm are typically associated with lower respiratory effects.

The patients exposed to Cl_2_ inhalation acutely, can appear in different range of clinical symptoms from fatal asphyxia or severe acute respiratory syndrome to slight injury such as simple irritation of the conjunctivae or nasal mucosa [1,8,12]. However, the lungs can also be permanently affected and clinically manifested as a chronic suppurative cough, complicated by wheezes and breathlessness of variable intensity [13,14].

In many reports the most prevalent complaints of intoxication cases, as reported in Sexton and Pronchik’s study [21], were skin, eye and throat irritations including pruritus, lacrimation, rhino rhea, conjunctiva irritation, oropharyngeal pruritus and irritation, cough, sore throat, laryngeal edema, dyspnea, and sometimes hoarseness and stridor, headache, chest pain, and anxiety [3, 4, 6, 9, 10, 12-15, 19, 22]. In our series most prevalent symptoms were cough (alone) and cough together with dyspnea. Cough and dyspnea mostly accompanied one another.

Agabiti et al [15] reported that eye irritation was observed in 50.0%, nose and throat problems in 54.5%, and respiratory symptoms in 71.6% of the children in their study group. The corresponding values among adults were reported as 61.9, 73.0, and 66.7% respectively. Moulick et al [12] noted that 42% of the patients had eye irritation, 29.2% headache, 26.8% abdominal pain, and 24.3% vomiting. Guloglu et al [9] had found 60.4% dyspnea and 17% cough and dyspnea in their series.

The authors did not find any skin or severe eye irritation in the patients. But respiratory and gastrointestinal symptoms were similar with the literature.

Physical examination following exposure to high concentrations may reveal decreased breath sounds, tachypnea, tachycardia, hypoxia, wheezing, crackles (ARDS/noncardiogenic pulmonary edema) and crepitation (associated with pneumomediastinum) [23]. In previous studies, Guloglu et al [9] reported that; 27.4% of their patients had expiratory wheezing, and only one had expiratory wheezing, cyanosis, tachycardia, and extrasystoles, 71.6% were normal. In our series, physical examination findings, normal patients’ ratios, and results of ABG analyses were similar. However, ECG’s, CXR findings were normal contrary to the literature.

A baseline CXR should be obtained if the patient is symptomatic and respiratory functions should be monitored, including ABG and pulse oxymetry [17]. Chest radiographs can be normal, or they can sometimes show diffuse nodular opacities, patchy consolidation, pulmonary edema, and signs of vascular congestion. Radiographic abnormalities may appear late as lung injury develops and progresses. Persistent hyper reactivity and airflow obstruction may manifest radiographically as air trapping. The role of computerized tomography in evaluating lung injury is not established [24].

The priority in acute exposures to high doses of Cl_2_ should be removal of person from the hazardous environment [5,17,25]. The goal of decontamination is to decrease further exposure to victims and to prevent secondary contamination of health care workers [26]. Immediate treatment of exposure, as with any toxic inhalation, should focus on the airway [25,26]. Patient with significant ocular symptoms (e.g., pain, photo-phobia, and vision changes) should have their eyes irrigated with plenty of water or saline solution. The eyes should be examined for corneal abrasions [17,26]. Also, for dermal injuries are caused by the caustic nature of the chlorine gas, contaminated clothing should be removed, followed by irrigation with plenty of water [5,17,26].

Guloglu et al [9] reported that they preferred and recommended humidified O_2_ and β agonist combination applications to be the supportive therapy in their study group because of trachea-bronchitis and broncho-constriction and/or pulmonary edema and Reactive Airway Dysfunction Syndrome effects of chlorine gas. Treatment implication was same in Akdur et al [14] case report of a 26 years old woman who was diagnosed as pneumomediastinum due to chlorine intoxication. They also used methyl-prednisolone and death was not reported. In 282 cases reported by Agabiti et al [15], intravenous cortisone and humidified O_2_ were used in some cases, particularly in those admitted to hospital. No patient received nebulized sodium bicarbonate treatment.

A few studies have used animal models, which involved systemic or inhaled corticosteroid administration treatment immediately following high-rate chlorine exposure. Gunnarsson et al [3] used a porcine model of Cl_2_ inhalation and demonstrated that the immediate use of inhaled beclomethasone improved hypoxemia, pulmonary vascular resistance, and survival. In a similar study with ventilated pigs, Wang et al [27] reported that the timing of inhaled corticosteroids may be an important factor in the management of chlorine intoxication. The same authors [3,27] demonstrated that the combination of an inhaled β_2_ agonist followed by a nebulized corticosteroid provided a greater improvement in Cl_2_-induced lung injury than the administration of each agent alone. The above studies showed improved pulmonary and cardiovascular function but no effect on mortality [3,27].

Nebulized sodium bicarbonate (NaHCO_3_) has also been suggested as a therapy for chlorine exposure. The proposed mechanism of the action is the neutralization of the formed acids in the respiratory tissues. Cellular damage from oxygen free radical formation is not addressed by this therapy [17,26,28,29]. Bosse GM [28] reported in his retrospective case review of nebulized NaHCO_3_ treatment of chlorine intoxication that 45.58% of patients clearly improved after using of bicarbonate. But there are not enough animal experiments about nebulized NaHCO_3_ therapy which will show the efficacy.

Against all, Russell et al [30] reported that there was no specific antidote for the treatment of casualties caused by exposure to chlorine, phosgene, or mustard; therefore, management is largely supportive. He also reported that, clinical data on corticosteroid treatment efficacy, which has been given in casualties because of accidental exposure to chlorine; were inconclusive because of the numbers of patient were inefficient and the indications for administration were unclear. Similarly, Fleta et al. [13] reported that the criteria for administration of treatment are not clear and thus the efficacy of treatment can not be ascertained.

Supplemental humidified oxygen, intravenous corticosteroids, bronchodilator combination and symptomatic supportive therapy were the main treatment choices in our series, similarly to the most of the literature. Applying too much steroids in our series can be a result of arbitrary decisions. Also, there is no scientific evidence for empiric use of antibiotics.

Patients with acute lung injury and/or upper airway burns may require endotracheal intubation and mechanical ventilation. Positive end expiratory pressure may be useful in enhancing oxygenation. Since pulmonary edema may be delayed, patients with significant symptoms should be admitted for observation and further symptomatic care for 24 hours. Asymptomatic patients may be discharged home with close follow up [17].

Based on published reports, most patients who are exposed to an isolated Cl_2_ inhalation, recovered completely [16,27]. However, noncardiogenic pulmonary edema, obstructive disease, restrictive disease, ARDS, pneumomediastinum has been reported in survivors [1, 8, 12-16].

Evans [16] had reported in his review that the reasons of variable outcomes of individuals were high level exposure, minute ventilation, and host characteristics such as pre-existent asthma or ongoing smoking.

In our series, in spite of different or arbitrary treatment modalities, there were no deaths and all patients’ symptoms and physical examination findings were resolved. This result, in spite not being very explanatory, may be an effect of early systemic steroid treatment.

### Study limitations

This study revealed the first most crowded accidental chlorine gas exposure in Turkey. Therefore authors could not find enough similar national study to compare. The patients were classified into three groups, but couldn’t compare in term of determine the significant differences such variables as exposure rate and age groups, and exposure rate and gender, because the reasons of groups’ having small patient number and don’t have heterogeneity and number similarity among groups.

Also, all patient treatments were not designed based on the literature, they were accepted completely arbitrary and authors could not classify the patients according to received treatments.

The important part of patients didn’t present for follow up because of socio-cultural reasons. This aspect affected our ability to gather information about long term prognosis. Furthermore, authors were not able to reach the patients medical data, who presented to other Ministry of Health Government Hospitals. We are not also aware of patients who were thought to be normal and had not been transferred from the scene of the accident by EMS. All of these can be the important factors in statistical and outcome evaluation.

### Conclusions

All exposed individuals in authors’ study have shown complete resolution of their symptoms. The authors recommend that non symptomatic or slightly exposed patients do not need any treatment or may be treated symptomatically. Combined humidified oxygen and bronchodilators are still the best treatment option in addition to symptomatic supportive measures. Animal experiments which research efficacy of different agents and their combinations on chlorine toxicity are necessary.

## References

[1] Lewis SN. Simple asphyxiants and pulmonary irritants. In: Goldfrank LR, Flomenbaum N, Hoffman RS, Howland MA, Lewin NA, Lewis SN, eds. Goldfrank’s Toxicologic Emergencies. 8th ed., New York: McGraw-Hill Medical Pub. Division; 2006. pp: 1673-688..

[2] Nemery B, Hoet PH, Nowak D (2002). Indoor swimming pools, water chlorination and respiratory health. Eur Respir J.

[3] Gunnarsson M, Walther SM, Seidal T, Lennquist S (2000). Effects of inhalation of corticosteroids immediately after experimental chlorine gas lung injury. J Trauma.

[4] Martinez TT, Long C (1995). Explosion risk from swimming pool chlorinators and review of chlorine toxicity. J Toxicol Clin Toxicol.

[5] Winder C (2001). The toxicology of chlorine. Environ Res.

[6] Traub SJ, Hoffman RS, Nelson LS (2002). Case report and literature review of chlorine gas toxicity. Vet Hum Toxicol.

[7] Wood BR, Colombo JL, Benson BE (1987). Chlorine inhalation toxicity from vapors generated by swimming pool chlorinator tablets. Pediatrics.

[8] Deschamps D, Soler P, Rosenberg N, Baud F, Gervais P (1994). Persistent asthma after inhalation of a mixture of sodium hypochlorite and hydrochloric acid. Chest.

[9] Guloglu C, Kara IH, Erten PG (2002). Acute accidental exposure to chlorine gas in the Southeast of Turkey: a study of 106 cases. Environ Res.

[10] Donnelly SC, FitzGerald MX (1990). Reactive airways dysfunction syndrome (RADS) due to chlorine gas exposure. Ir J Med Sci.

[11] Shusterman DJ, Murphy MA, Balmes JR (1998). Subjects with seasonal allergic rhinitis and nonrhinitic subjects react differentially to nasal provocation with chlorine gas. J Allergy Clin Immunol.

[12] Moulick ND, Banavali S, Abhyankar AD, Borkar S, Aiyengar J, Kapadia NM, Khokhani RC (1992). Acute accidental exposure to chlorine fumes--a study of 82 cases. Indian J Chest Dis Allied Sci.

[13] Fleta J, Calvo C, Zuniga J, Castellano M, Bueno M (1986). Intoxication of 76 children by chlorine gas. Hum Toxicol.

[14] Akdur O, Durukan P, Ikizceli I, Ozkan S, Avsarogullari L (2006). A rare complication of chlorine gas inhalation: pneumomediastinum. Emerg Med J.

[15] Agabiti N, Ancona C, Forastiere F, Di Napoli, Lo Presti, Corbo GM, D'Orsi F (2001). Short term respiratory effects of acute exposure to chlorine due to a swimming pool accident. Occup Environ Med.

[16] Evans RB (2005). Chlorine: state of the art. Lung.

[17] Howard C, Ducre B, Burda AM, Kubic A (2007). Management of chlorine gas exposure. J Emerg Nurs.

[18] Ruckart PZ, Wattigney WA, Kaye WE (2004). Risk factors for acute chemical releases with public health consequences: Hazardous Substances Emergency Events Surveillance in the U.S., 1996-2001. Environ Health.

[19] Mrvos R, Dean BS, Krenzelok EP (1993). Home exposures to chlorine/chloramine gas: review of 216 cases. South Med J.

[20] D'Alessandro A, Kuschner W, Wong H, Boushey HA, Blanc PD (1996). Exaggerated responses to chlorine inhalation among persons with nonspecific airway hyperreactivity. Chest.

[21] Sexton JD, Pronchik DJ (1998). Chlorine inhalation: the big picture. J Toxicol Clin Toxicol.

[22] Decker WJ (1988). Chlorine poisoning at the swimming pool revisited: anatomy of two minidisasters. Vet Hum Toxicol.

[23] Das R, Blanc PD (1993). Chlorine gas exposure and the lung: a review. Toxicol Ind Health.

[24] Kanne JP, Thoongsuwan N, Parimon T, Stern EJ (2006). Trauma cases from Harborview Medical Center. Airway injury after acute chlorine exposure. AJR Am J Roentgenol.

[25] Vohra R, Clark RF (2006). Chlorine-related inhalation injury from a swimming pool disinfectant in a 9-year-old girl. Pediatr Emerg Care.

[26] White SR, Eitzen CE, Klein KR. Toxicology of Hazardous Chemicals. In: Tintinalli JE, Kelen GD, Stapcyznski JS, eds. Emergency Medicine. A Comprehensive Study Guide. 6th Ed. New-York: McGraw-Hill Co; 2004. p. 1153-160..

[27] Wang J, Zhang L, Walther SM (2002). Inhaled budesonide in experimental chlorine gas lung injury: influence of time interval between injury and treatment. Intensive Care Med.

[28] Bosse GM (1994). Nebulized sodium bicarbonate in the treatment of chlorine gas inhalation. J Toxicol Clin Toxicol.

[29] Aslan S, Kandis H, Akgun M, Cakir Z, Inandi T, Gorguner M (2006). The effect of nebulized NaHCO3 treatment on "RADS" due to chlorine gas inhalation. Inhal Toxicol.

[30] Russell D, Blain PG, Rice P (2006). Clinical management of casualties exposed to lung damaging agents: a critical review. Emerg Med J.

